# Strengthening Primary Care With a Minimal Digital Ecosystem in Burkina Faso: Protocol for a Pragmatic Mixed Methods Implementation Study

**DOI:** 10.2196/86135

**Published:** 2026-05-22

**Authors:** David Zombré, Joël Arthur Kiendrébéogo, Issa Kaboré, Simon Tiendrébéogo, Yamba Kafando, Michael Chaitkin, Rémi Kaboré, Charlemagne Tapsoba, Orokia Sory, Nacanabo Relwendé, Noellie Konsebo, Mamadou Sani Taram, Boureima Paré, Wend-Timbe-Noma Arlette Raïssa Zongo, Daliah Marie Nacoulma, Wendyiida Bibata Derra, Abdoul Razak Hebie, Pierre Yaméogo

**Affiliations:** 1 Recherche Pour la Santé et le Développement (RESADE), Secteur 28, Ouagadougou, 04 BP 8398, Burkina Faso, 226 73621620; 2 Université Joseph Ki-Zerbo, Ouagadougou, Centre, Burkina Faso; 3 Ministère De La Santé, Ouagadougou, Burkina Faso; 4 Institut de Recherche en Sciences de la Santé (IRSS), Ouagadougou, Burkina Faso; 5 Global Development Division, Gates Foundation, Seattle, WA, United States; 6 Bordeaux School of Public Health (ISPED), University of Bordeaux, Bordeaux, France; 7 Faculty of Nursing, VITAM – Centre de recherche en santé durable, Quebec, QC, Canada

**Keywords:** implementation science, digital health, primary health care, Burkina Faso, CFIR, NPT, mixed methods, scale validation, Consolidated Framework for Implementation Research, Normalization Process Theory

## Abstract

**Background:**

In Burkina Faso, the Minimal Digital Ecosystem (MDE)—a suite of 9 integrated digital tools—was introduced to support key health system functions, including care delivery, financial management, medication oversight, governance, and data use. However, evidence regarding the maturity of its real-world implementation and the determinants influencing its adoption remains scarce.

**Objective:**

This pragmatic mixed methods study aims to (1) measure MDE implementation maturity across 4 dimensions (adoption, fidelity, penetration, sustainability), (2) identify multilevel determinants using the Consolidated Framework for Implementation Research (CFIR 2.0) and Normalization Process Theory (NPT), and (3) examine associations between implementation degree and primary health care (Centre de Santé et de Promotion Sociale [CSPS]) performance

**Methods:**

We use a sequential explanatory design (quantitative → qualitative) in 4 districts covering 72 CSPSs. Phase 1 involves a cross-sectional survey of all eligible health workers, facility managers, and community health workers (estimated 612 respondents nested within facilities) using CFIR- and NPT-informed questionnaires. Following psychometric validation (exploratory/confirmatory factor analysis; reliability assessment via Cronbach α and McDonald ω), we will fit multilevel models with CSPS random intercepts and district fixed effects to (1) quantify between-facility implementation variance, (2) test associations with CFIR and NPT determinants, and (3) examine relationships with CSPS performance indicators. Phase 2 involves purposive sampling of facilities with varying implementation profiles for interviews, focus groups, and observations, analyzed using reflexive thematic analysis to explain quantitative patterns.

**Results:**

Ethics approval was obtained from Burkina Faso’s National Ethics Committee (number 2023-06-136). The study was funded in June 2022 (Gates Foundation, Grant INV-056021) and is being conducted across 72 primary health care facilities in 4 districts (Manga, Sapouy, Ténado, and Ziniaré) in Burkina Faso. Qualitative data collection commenced in November 2025 and was completed in January 2026. Quantitative data collection took place from January 2023 to October 2025. As of May 2026, qualitative data collection was completed, and quantitative analyses had commenced. Psychometric analyses are underway.

**Conclusions:**

By addressing key evidence and measurement gaps in digital health implementation, this protocol will (1) generate context-specific guidance on implementing and institutionalizing a complex digital health ecosystem and (2) provide validated, ready-to-use instruments to quantify implementation at the CSPS level. Together, these outputs will help policymakers and program managers define and track “implementation success,” informing—and accelerating—the scale-up and optimization of the MDE in resource-constrained settings.

## Introduction

### Background

Modernizing health systems with digital technologies offers a practical path to tackle persistent primary health care (PHC) challenges across sub-Saharan Africa (SSA) [1]. Adoption of digital innovations—from mobile health (mHealth) apps to electronic health records—is accelerating and has the potential to improve service delivery, data quality, and access to care, particularly for vulnerable populations [2,3]. However, fragmentation across digital tools can hinder strategic planning, monitoring and evaluation, and data-driven decision-making [4,5]. Real-world deployment also faces substantial barriers, including infrastructure gaps, limited digital literacy among health care workers, and resource constraints [6,7], which create a persistent gap between the promise of digital health and its system-level impact [6,7]. In Burkina Faso, where Primary Health and Social Promotion Centers (Centres de Santé et de Promotion Sociale [CSPSs]) form the backbone of the health system [8], the Minimal Digital Ecosystem (MDE) was introduced to strengthen service delivery, health financing and financial management, medicines oversight, governance, and data use while supporting the national free-care policy (gratuité) [9]. The MDE is not a single application but a set of integrated tools spanning point-of-care and managerial functions (eg, electronic consultation registers, child and maternal care modules, individual electronic care records/Fiche Individuelle de Soins [individual care sheets], free care management, stock and financial management, quality assessment, and community health platforms) [9]. Given this complexity, a rigorous evaluation of implementation is essential for understanding the determinants of success and guiding sustainability and scale-up.

This study addresses two critical gaps in the research on digital health implementation. First, validated, standardized instruments to measure the maturity of complex digital health ecosystems in resource-constrained settings remain scarce [10,11]. Although frameworks such as the Consolidated Framework for Implementation Research (CFIR) and Normalization Process Theory (NPT) enumerate potential determinants, few tools enable their quantification and examination alongside key implementation outcomes (adoption, fidelity, penetration, sustainability) [10,12]. Second, this measurement gap creates a translational challenge: Without evidence-based benchmarks, policymakers cannot define “implementation success” to target support or guide scaling decisions. In Burkina Faso, the widespread, nonrandom rollout of the MDE precluded a traditional quasi-experimental design but created a pragmatic opportunity to answer a stakeholder question from the Ministry of Health: *At what threshold can a PHC facility be considered to have fully implemented the MDE?* This underscores the need for an evidence-based, measurable definition of “implementation success” to inform resource allocation and policy [10]. Using a pragmatic, mixed methods design [13], this protocol aims to generate a contextualized understanding of MDE implementation, identify levers for optimization, and produce validated instruments to assess implementation maturity—evidence intended to inform digital health policy in Burkina Faso and similar resource-constrained settings.

### Study Goals and Objectives

The MDE evaluation was initially structured into 5 complementary surveys across its implementation stages [9]. The initial phase profiled users, analyzed the implementation, and evaluated the early effects; the second explored the deployment context, including resources, preparedness, user perceptions, and the free access application; and the third assessed deployment levels, utilization, satisfaction, service governance, and quality. Although this multiphase approach provided foundational data, the objectives for the ongoing fourth survey and the prospective fifth were substantively revised in response to real-world implementation challenges that necessitated a shift in the evaluation’s focus.

### Defining the “Pragmatic” Approach

We adopted a pragmatic paradigm [14], an approach in implementation science that prioritizes useful, real-world solutions over theoretical purity. We aim to generate actionable knowledge in the complex setting of actual practice, not to test a hypothesis under perfect, controlled conditions. Our approach is pragmatic in 3 specific ways.

First, from a methodological standpoint, we embrace flexibility in response to field realities. When intervention contamination threatened the validity of our original quasi-experimental design, we shifted to an implementation-focused, cross-sectional observational evaluation better suited to the real-world context.

Second, from an epistemological standpoint, we integrate multiple frameworks (CFIR 2.0, NPT) and methods (quantitative surveys, qualitative inquiry). We recognize that no single approach can adequately answer the complex questions our stakeholders have.

Third, contextually, we are evaluating the MDE “as deployed” rather than under idealized conditions. We treat the natural variation in implementation not as statistical “noise” to be controlled but as a rich source of insight into what is actually happening on the ground.

This pragmatic orientation ensures that our findings will be directly relevant to policymakers and program managers navigating the complexities of scaling up digital health in resource-constrained settings.

### Justification for the Revised Evaluation Objectives

Initially, our evaluation plan for the MDE was a quasi-experimental design. We intended to compare two intervention districts (Ténado and Ziniaré) with two control districts (Manga and Sapouy) to assess the ecosystem’s impact. However, the operational reality of field deployment necessitated a significant deviation from this a priori approach.

However, a key challenge emerged early on: The MDE tools began to progressively and nonrandomly diffuse into our designated control districts. This “contamination” effect fundamentally compromised our original quasi-experimental design—a common problem in real-world implementation studies.

As a result, a robust, direct comparison between the groups became untenable, making it impossible to isolate the MDE’s specific effects. This cross-contamination severely threatened the study’s internal validity; we could no longer confidently attribute observed outcomes to the intervention and faced a high risk of biased or artificially weakened (attenuated) effect sizes.

Consequently, we reoriented the focus of the evaluation. Rather than pursuing a simple effectiveness question, our approach now aligns more closely with an implementation science perspective, which acknowledges and addresses the complexities of real-world contexts [14].

This real-world deployment scenario has raised a critical question from Ministry of Health stakeholders: At what threshold of adoption and routine use can a health facility be considered to have fully implemented the MDE initiative? This new question aligns perfectly with the field’s focus on understanding and measuring implementation outcomes—the direct effects of actions taken to implement an innovation, which serve as key indicators of success [10].

To address both the contamination challenge and this new, emergent research question, we adapted our methodology. Instead of a rigid comparative analysis, we are now using a pragmatic, integrated evaluation design. This shift ensures our evaluation remains relevant and provides actionable insights for future scale-up [15].

Our revised focus will assess the varying levels of MDE tool adoption (or “dose”), its integration into clinical and management workflows, and the maturity of its use across all districts. We will adjust our analytical approach to explore the relationship between these implementation outcomes and health service outcomes [10]. This will allow us to better understand the associations and mechanisms, even in the context of widespread tool availability.

Ultimately, this approach will provide stakeholders with more nuanced and actionable insights into how to optimize MDE implementation to achieve greater impact on the health system.

Specifically, this study aims to (1) assess the maturity of MDE implementation, (2) identify multilevel determinants, and (3) evaluate the association between MDE implementation and health facility outcomes.

To assess the maturity of MDE implementation, we will evaluate the level of adoption; fidelity to its intended use; penetration into existing practices, perceived sustainability, and institutionalization of its various tools. We will use the NPT framework to guide our understanding of institutionalization.

To identify multilevel determinants, we will use the CFIR 2.0 to identify the facilitators and barriers that influence the degree and quality of MDE implementation. This will encompass a broad range of factors, including intervention characteristics, outer and inner settings, individual user characteristics, and the implementation process itself.

To evaluate the association between MDE implementation and health facility outcomes, we will assess the relationship between the degree of MDE implementation and the readiness and quality of health facility services. This evaluation will particularly focus on key areas such as service delivery, financial and medication management, the efficiency of free health care provision, and the use of data for decision-making.

### Hypotheses

Building on the study’s objectives to evaluate the implementation and effects of the MDE, we propose the following 4 hypotheses to guide our investigation.

Hypothesis 1 (H1) involves implementation maturity variance. We hypothesize that the maturity of MDE implementation will vary significantly across different MDE tools, CSPS, and the districts. This reflects the complexity of real-world implementation contexts.

H2 involves multilevel determinants. We hypothesize that the degree and quality of MDE implementation are associated with multilevel determinants. Specifically, positive factors at the inner setting level (eg, engaged leadership, adequate resources, a favorable implementation climate) and within the implementation process (eg, quality of training, ongoing support) will be positively correlated with a higher degree and better quality of MDE implementation.

H3 involves user perceptions and adoption. We hypothesize that the degree of MDE implementation is associated with user perceptions of the intervention’s characteristics. A positive perception of MDE characteristics (eg, relative advantage, low complexity) by individual users will be positively correlated with higher adoption and utilization rates of MDE tools, contributing to a greater overall degree of implementation.

H4 involves implementation and facility outcomes. We hypothesize that a higher degree of MDE implementation will be positively associated with improved health facility readiness and performance. This association will be particularly evident in enhanced service delivery, more efficient financial and medication management, better management of free health care services, and increased utilization of data for informed decision-making within CSPS.

## Methods

### Study Setting

This study is being conducted in 4 health districts in Burkina Faso: Ziniaré (Plateau Central region), Ténado and Sapouy (Centre-Ouest region), and Manga (Centre-Sud region). Although these districts were initially selected for a quasi-experimental design [16], the widespread, real-world deployment of the MDE has reframed the study. This collection of districts now serves as a setting with valuable natural variation in implementation levels [17], allowing us to assess varying levels of MDE implementation and identify the multilevel determinants influencing its adoption and institutionalization across diverse settings.

### The MDE in Context: Novelty and Distinctiveness

The MDE represents a novel approach to digital health implementation in SSA. Unlike standalone digital interventions, such as isolated electronic medical record systems or single-purpose mHealth apps, the MDE deliberately integrates 9 complementary tools into a cohesive ecosystem spanning clinical care, supply chain management, financial tracking, quality assurance, and community health [9]. The “minimal” designation reflects a strategic choice: Rather than implementing a comprehensive, resource-intensive hospital information system, Burkina Faso’s Ministry of Health prioritized essential, interoperable tools that can be deployed at the PHC level within existing infrastructure [9]. This ecosystem model has not been systematically evaluated elsewhere in SSA, making this study among the first to rigorously assess the implementation of an integrated digital health suite—rather than discrete applications—in a low-resource setting. Additionally, although Burkina Faso has previously piloted individual digital health tools (eg, DHIS2 for health information, isolated mHealth interventions), the MDE’s distinguishing feature is the intentional harmonization and codeployment of multiple tools, designed to generate synergies in data flow, reduce parallel workflows, and support holistic PHC strengthening [9].

The inclusion of all 4 districts enables the study to leverage their distinct characteristics—such as urban/rural profiles, population density, and pre-existing digital infrastructure—to robustly address the implementation-focused research objectives. This ensures a comprehensive evaluation that considers a broad range of contexts.

### Intervention Description

**Figure 1.**
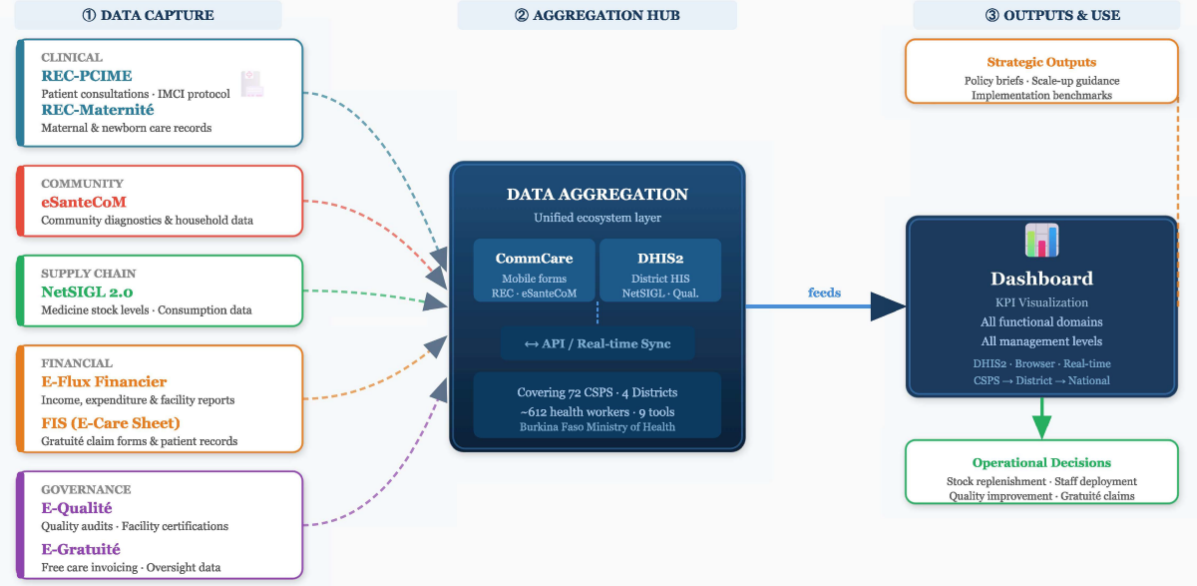
The Minimal Digital Ecosystem (MDE) as an integrated tool architecture with 9 complementary digital tools deployed across primary health care facilities in Burkina Faso, converging on a central decision-support dashboard. Tools are color-coded by functional domain: clinical care (teal), financial management (orange), supply chain (green), governance and quality (purple), community health (red), and decision support (navy). CommCare: mobile data collection platform; CSPS: Centre de Santé et de Promotion Sociale; DHIS2: District Health Information System; FIS: Fiche Individuelle de Soins (Individual Care Sheet); IMCI: Integrated Management of Childhood Illness; KPI: key performance indicator; MDE: Minimal Digital Ecosystem.

The intervention under evaluation is the MDE, a suite of integrated digital tools designed to strengthen PHC in the 4 study districts of Burkina Faso. The MDE aims to enhance operational efficiency, service quality, governance, and data-driven decision-making by harmonizing various digital applications into a cohesive system. The core tools of the MDE are detailed in Table 1 and Figure 1.

**Table 1.** Key characteristics of the selected study districts in Burkina Faso, sourced from the 2023 Statistical Yearbook of the Ministry of Health.

Characteristic	Ziniaré	Ténado	Manga	Sapouy
Administrative region				
Name of the administrative region	Plateau-Central	Centre-Ouest	Centre-Sud	Centre-Ouest
Classification	Peri-urban	Rural	Peri-urban	Rural
Total population, n	345,471	211,543	340,912	267,015
Health facilities, n	72	28	48	33
Key health indicators				
Facility-based delivery rate, %^a^	102.2	95.6	111.3	103.6
ANC4^b^ visit rate, %	52.7	65.4	51.5	30.3
IPTp3^c^ rate, %	73.9	70.1	70.4	54.7
Neonatal mortality rate (per 1000), n	2.3	—^d^	14	6
Maternal mortality ratio (per 100,000)	14.5	0	25	51.8

a Facility-based rates >100*%* may be present due to using projected population denominators or to the inflow of patients from other districts.

b ANC4: antenatal care - at least four visits.

c IPTp3: pregnant women attending antenatal care at least once and receiving at least 3 doses of Intermittent Preventive Treatment of Malaria for Pregnant Women.

d Not applicable.

This study is not an interventional clinical trial; however, the MDE is a complex intervention for which the implementation is being evaluated. Accordingly, we adapted the SPIRIT 2013 guidance to structure the protocol, particularly the sections describing the intervention, objectives, outcomes, target population, and analysis plan. We also completed the TIDieR (Template for Intervention Description and Replication) checklist to enable precise reporting of interventions. The finalized checklists are provided as Checklist 1.

### MDE Intervention Theory of Change

The MDE’s theory of change posits that core health system functions will be digitized and streamlined by deploying an integrated suite of digital tools and supporting their use through targeted training. This is expected to improve clinical protocol adherence, financial and supply chain efficiency, and systematic data collection at the facility and community levels. By aggregating this newly captured, real-time data into a central dashboard, the MDE then aims to foster evidence-based decision-making among managers. The ultimate goal of this combined improvement in service quality, operational efficiency, governance, and data utilization is to produce a modernized and strengthened PHC system capable of delivering higher-quality and more equitable care.

### Study Design

This study will use a cross-sectional mixed methods design [18] guided by a pragmatic approach [14]. This design is particularly well-suited to our revised objectives, as it allows us to pivot from a rigid comparative analysis to an observational one, an ordinary and necessary adjustment when researchers cannot fully control interventions in real-world settings [19]. The design will be implemented in two distinct phases where the quantitative findings inform the subsequent qualitative inquiry.

### Phase 1: Quantitative

The first phase of the study will consist of a cross-sectional quantitative survey designed to address both the primary and secondary objectives. This phase will assess the extent of MDE implementation across all participating health facilities and districts. To achieve this, we will quantify key implementation outcomes based on the CFIR, with a specific focus on adoption, fidelity, penetration, and sustainability. The findings will support the development of a typology of implementation profiles, which will inform and guide the strategic deployment of a digital health ecosystem in Burkina Faso (Table 2).

We will estimate the associations between the implementation level and (1) multilevel determinants specified by CFIR 2.0 and NPT and (2) key health facility performance indicators using appropriate multivariable models.

This phase will answer the “what” and “how much” questions, providing a broad overview of implementation maturity and identifying the significant relationships outlined in our hypotheses (H1-H4).

**Table 2.** Summary of digital health tools: purpose, users, and technology platforms structured according to the TIDieR (Template for Intervention Description and Replication) checklist to ensure a complete and standardized characterization.

Tool name	Core function and purpose	Primary users and technology
REC^a^-PCIME^b^	Guides child health consultations using the IMCI^c^ protocol; creates electronic patient records	Health workers (tablet, CommCare^d^)
REC-Maternité	Guides maternal and newborn care (ANC^e^, delivery, PNC^f^, family planning); creates electronic records	Health workers (tablet, CommCare)
E-Flux Financier	Monitors real-time financial data (income, expenditure) and automates facility financial reports.	Financial managers (tablet)
FIS^g^	Digitizes patient care sheets for the national free health care (gratuité) program; serves as a claim form	Health workers (tablet)
NetSIGL 2.0	Manages real-time data on medicine and supply stocks across the health system	Health workers (tablet, DHIS2^h^)
eSanteCoM	Provides community-level diagnostic and treatment guidelines and data collection for community health workers (ASBCs^i^)	ASBCs (phone, CommCare)
E-Qualite	Manages digital checklists for internal and external quality audits and facility certification processes	Facility managers (tablet/computer, DHIS2)
E-Gratuité	Collects and monitors data for the national gratuité scheme, including invoicing, payments, and workload; follows up/provides oversight of medical devices and equipment	Facility and district managers (tablet/computer, DHIS2)
Dashboard	Visualizes and integrates key performance indicators from all MDE^j^ tools to support decision-making	Health managers at all levels (computer/browser)

a REC: Registre Électronique de Consultation (Electronic Consultation Register).

b PCIME: Prise en Charge Intégrée des Maladies de l’Enfant (Integrated Management of Childhood Illness).

c IMCI: Integrated Management of Childhood Illness.

d CommCare: mobile data collection platform.

e ANC: antenatal care.

f PNC: prenatal care.

g FIS: Fiche Individuelle de Soins (Individual Care Sheet).

h DHIS2: District Health Information System.

i ASBC: Agent de Santé à Base Communautaire.

j MDE: Minimal Digital Ecosystem.

### Phase 2: Qualitative

The second phase will consist of a qualitative inquiry designed to explain and elaborate upon the findings from the quantitative phase. Using a multiple-case study approach, we will purposively select a subset of health facilities that demonstrate particularly high, low, or mixed levels of implementation based on the quantitative results. This phase will explore the “how” and “why” behind the statistical patterns through semistructured interviews, focus groups, and direct observation. For example, it will help us understand why specific MDE tools are well-integrated while others are not, how specific facilitators (eg, leadership engagement) operate in practice, and what contextual barriers hinder the translation of the MDE into improved performance.

### Theoretical Frameworks

#### Consolidated Framework for Implementation Research (CFIR 2.0)

We will primarily use CFIR 2.0 to assess the extent of MDE implementation and its determinants [12]. CFIR 2.0 provides a structured approach by categorizing determinants into 5 key domains: intervention, outer setting, inner setting, individuals, and process. Crucially, its Outcomes Addendum links these implementation processes to their results, addressing a key need in the field [12]. This is particularly relevant, as it directly links these implementation processes to their results, which is a critical need in the field. We will use this framework to analyze CFIR-defined contextual factors as potential moderators of the MDE’s degree of implementation. The strength of CFIR 2.0 lies in its ability to concurrently evaluate the degree of implementation and its determinants, which helps to clarify the relationships between them [12].

#### Normalization Process Theory (NPT)

Complementarily, we will use NPT [20] to analyze how MDE tools are integrated and institutionalized into daily practices. NPT focuses on the collective work required for a new practice to become routine, examining it through 4 key constructs: coherence, cognitive participation, collective action, and reflexive monitoring [20]. NPT’s specific relevance to our evaluation is its ability to provide a nuanced understanding of how MDE tools become—or fail to become—normalized and sustained within the complex daily workflows of health care providers. Although CFIR helps identify the factors that influence implementation, NPT will illuminate the social dynamics and processes that embed these tools in routine practice. Applying NPT will enable a precise analysis of the mechanisms driving the integration and institutionalization of MDE tools by examining collective sense-making, user engagement, practical operationalization, and iterative appraisal and adaptation processes [20].

### Population and Selection Criteria

#### Recruitment and Sampling Procedures

##### Quantitative Phase: Sample Size Justification and Composition

###### Population

This study will engage a multilevel population across the 4 health districts. We selected 72 PHCs (J=72) across 4 districts and will conduct a census of all eligible cadres within each facility (health care providers, Infirmiers Chefs de Poste [ICPs; facility managers], or chief doctors), pharmaceutical depot managers, facility management committee treasurers, and community health workers (Agent de Santé à Base Communautaire [ASBCs]). Based on comprehensive staffing audits conducted during study preparation, the sample composition is detailed in Table 3.

This configuration yields a total expected sample of 612 respondents (range of 585 to 640 depending on ASBC availability and staff turnover). The sample size was determined through the considerations outlined in the following sections.

**Table 3.** Sample composition by district and cadre.

District	CSPS^a^, n	ICPs^b^, n	Providers^c^, n	Depot managers, n	Treasurers, n	ASBCs^d^^,^^e^, n	Total per district, n
Ziniaré	20	20	60	20	20	60	180
Manga	20	20	60	20	20	60	180
Ténado	16	16	48	16	16	40	136
Sapouy	16	16	48	16	16	40	136
Total sample	72	72	216	72	72	200	612

a CSPS: Centre de Santé et de Promotion Sociale (Primary Health and Social Promotion Center)

b ICPs: Infirmier Chefs de Poste (Head Nurses of Post).

c Providers include nurses, midwives, and other clinical cadres using Minimal Digital Ecosystem (MDE) tools; estimated at 3 per facility on average based on staffing data.

d ASBCs: Agent de Santé à Base Communautaire (community-based health workers).

e Average 2.5-3 per facility catchment area; counts reflect active ASBCs trained on eSanteCoM.

###### Precision for Implementation Scores

With n=612, the margin of error for the primary continuous implementation score (assuming σ=1.0 on a standardized scale) is ±0.08 at 95% confidence—sufficient precision for characterizing implementation maturity and detecting meaningful facility-level variation.

###### Multilevel Modeling Requirements

The nested design (respondents within CSPS within districts) requires adequate sample sizes at each level:

For level 1 (individual), n=612 provides ample power for individual-level predictors. For level 2 (CSPS), J=72 facilities substantially exceed the minimum recommended threshold of 50 clusters for stable variance component estimation [21]. For level 3 (district), with only 4 districts, we will model the district as a fixed effect rather than a random effect, avoiding the instability associated with <10 clusters [22,23].

The mean cluster size of m≈8.5 respondents per facility creates a design effect of design effect (DEFF)=1+(m−1)× intracluster correlation coefficient (ICC). Under plausible ICC values informed by prior implementation research in SSA settings (ICC=0.05 to 0.20), the effective sample size remains adequate: ICC=0.05 → DEFF=1.375 → n_eff=445; ICC=0.10 → DEFF=1.75 → n_eff=350; ICC=0.20 → DEFF=2.50 → n_eff=245. All scenarios provide sufficient power for multilevel regression analyses.

###### Power for Primary Hypotheses

Power analyses were conducted using Optimal Design Plus software [24], assuming the following: primary outcome = standardized implementation score (mean=0, SD=1); 2-level model (individuals nested in facilities); target power=80% at *α*=.05 (2-tailed); and anticipated ICC=0.10 (conservative estimate based on similar digital health implementation studies).

Results indicate adequate power (>80%) to detect (1) small-to-medium associations (*r*≥0.15) between individual-level determinants and implementation scores, (2) medium facility-level effects (Cohen *d*≥0.40) when comparing facilities by key characteristics (eg, urban/rural, high/low leadership engagement), and (3) district differences of *d*≥0.50 using fixed-effects contrasts

###### Facility-Level Predictor Capacity

With 72 facilities and 4 districts, we can reliably estimate up to 28 to 30 facility-level predictors (following the guideline of at least 2.5 clusters per predictor) [21]. Our analysis plan includes approximately 12 to 15 facility-level covariates derived from CFIR domains (eg, organizational readiness, resource availability, leadership engagement), well within this limit.

###### Accounting for Attrition

We anticipate an 8% to 10% nonresponse rate due to extended leave, turnover, or refusal. Our target of 612 eligible respondents includes a 10% buffer, ensuring a minimum analyzable sample of n=550.

### Qualitative Phase: Purposive Sampling Strategy

Following quantitative analysis, we will use maximum variation sampling to select facilities representing the full implementation spectrum. Target qualitative sample sizes, informed by prior implementation research and saturation principles [25-27], are described in the following sections.

#### Semistructured Interviews

Semistructured interviews will be conducted with 24 to 30 participants carefully selected to represent multiple levels of the health system and community stakeholders. This will include 12 to 15 facility staff, such as infection control and prevention personnel, health care providers, and depot managers, drawn from 8 to 10 purposively selected primary health centers. Additionally, 8 to 10 key informants operating at the district, regional, and central levels will be interviewed, including members of Essential Childhood Disease committees and Medical Device Equipment focal points. Finally, 4 to 5 community representatives will participate, including community-based health worker supervisors and members of health facility management committees, ensuring that community perspectives are adequately captured during data collection.

#### Focus Group Discussions

Focus group discussions will be organized into 6 to 8 groups, with each group comprising 6 to 8 participants to facilitate in-depth collective dialogue and exchange of perspectives. The composition will include 2 to 3 provider groups, stratified by their level of implementation experience to capture varied insights across operational contexts. Two focus groups will be conducted with community-based health workers to understand frontline service delivery challenges and experiences. Additionally, 1 to 2 facility management groups will be convened to explore organizational and administrative perspectives on program implementation. Finally, one focus group will bring together district managers to examine coordination, supervision, and support mechanisms at the district level, ensuring comprehensive representation across different stakeholder categories within the health system.

Recruitment will continue until thematic saturation—the point at which no substantively new codes or themes emerge across two consecutive interviews or focus group discussions [26]. We anticipate saturation within the planned sample range based on the study’s focused scope and homogeneous professional populations.

### Outcomes and Data Collection

The study’s primary and secondary outcomes are defined and measured as described in the following sections.

#### Primary Outcomes

##### Degree of Implementation of MDE Tools

This outcome will be assessed across 4 key dimensions, aligned with implementation science frameworks (CFIR 2.0), including adoption, fidelity, penetration, and sustainability.

The adoption dimension will be assessed by examining the tools’ perceived usefulness and ease of learning, alongside their initial utilization rate.

Fidelity will be measured by evaluating the adherence to intended procedures and the completeness of data entered.

The penetration dimension will quantify the percentage of eligible activities covered by the tool and its integration into daily routines.

Sustainability will encompass the intention to continue use, the perception of the tool as standard practice, and the availability of ongoing support.

These dimensions will be measured using questionnaires tailored to each MDE tool and using Likert scales, multiple choice questions, and percentage estimates.

##### Implementation Score Calculation

To operationalize the degree of implementation, a multistep scoring methodology will be applied [28,29]. First, for data coding, responses to Likert-scale items will be numerically coded from 1 for “Strongly Disagree” to 5 for “Strongly Agree.” Any negatively worded items will be reverse-coded so that higher scores consistently indicate more positive implementation outcomes.

An overall implementation score will be calculated for each MDE tool in a 2-step process: (1) The mean score for all items within each of the 4 dimensions (Adoption, Fidelity, Penetration, Sustainability) will be calculated, and (b) these 4 dimension scores will then be averaged to generate a single, overall implementation score for that tool [10,12].

Finally, to create a composite score for the entire MDE, the mean of the individual tool scores will be calculated. A weighted average, where tools are assigned weights based on strategic importance, will also be explored as an alternative analytical approach to provide a more nuanced overall score. The tool-weighting exercise will be conducted with stakeholders (central Ministry of Health decision-makers, tool developers, and district and facility managers) using established approaches for participatory, multicriteria weighting [30-32]. All scale construction and scoring will follow good practice in measurement and psychometrics [33].

##### Determinants of Implementation

These factors will be identified and assessed using the 5 domains of CFIR 2.0: Intervention Characteristics, Outer Setting, Inner Setting, Characteristics of Individuals, and the Implementation Process. They will be measured primarily using the questionnaire administered to health center managers (ICPs) and through open-ended questions in the tool-specific questionnaires and the qualitative data.

##### Secondary Outcomes

The level of institutionalization will be measured using constructs from NPT, primarily via the “Sustainability” sections of the questionnaires, and explored in-depth through qualitative data.

The perceived impact of the MDE on quality of care, efficiency, governance, and user satisfaction will be explored qualitatively and via specific questions in the health center manager questionnaire.

### Measurement Instruments

Data for this study will be collected using a suite of mixed methods instruments designed to capture both the degree of MDE implementation and its underlying determinants.

#### Quantitative Instruments

Two primary structured questionnaires will be used for the quantitative phase.

For tool-specific implementation, a set of modular questionnaires will be administered to the direct users of each MDE tool (eg, REC-PCIME [Registre Électronique de Consultation - Prise en Charge Intégrée des Maladies de l’Enfant], NetSIGL). Each module is tailored to a specific tool and designed to measure the primary implementation outcomes (Adoption, Fidelity, Penetration, Sustainability) using a combination of Likert-scale items, multiple-choice questions, and direct estimates.

For the determinants of implementation, a second questionnaire, guided by the CFIR 2.0, will be administered to all facility managers (ICPs) and a sample of community-based health workers (CBHWs). This instrument assesses the multilevel determinants that influence implementation, such as organizational readiness, leadership engagement, and resource availability.

#### Qualitative Instruments

Semistructured interviews and focus group discussion guides will be developed for the qualitative phase. The CFIR and NPT will theoretically inform these guides. Critically, the guides will be finalized after the initial analysis of the quantitative data to ensure they specifically explore and explain key statistical findings, such as implementation challenges, performance variations between sites, or unexpected results.

### Scale Development and Validation

Prior to the main data analysis, a rigorous process will be undertaken to develop and validate the quantitative measurement scales from the questionnaire data [34]. This process applies to the composite implementation scores for each MDE tool and for the MDE overall, as well as to scales measuring key implementation determinants (eg, constructs from the CFIR framework).

We are evaluating the measurement properties in line with COSMIN (Consensus-Based Standards for the Selection of Health Measurement Instruments) guidance, covering content validity, structural validity, internal consistency, reliability, measurement error, and hypothesis-testing construct validity. Content validity was addressed through expert review and piloting (relevance, comprehensiveness, comprehensibility) [35]. For structural validity, we will first assess sampling adequacy (Kaiser-Meyer-Olkin≥0.70; Bartlett test *P*<.001) and conduct exploratory factor analysis (EFA) using polychoric correlations with minimum residual extraction and oblique rotation; item retention requires primary loading ≥0.40, cross-loading difference ≥0.30, and communality ≥0.30. We will then run confirmatory factor analysis (CFA) using weighted least squares mean and variance adjusted for ordinal Likert items (or maximum likelihood/maximum likelihood robust estimation if distributions are approximately continuous), reporting fit indices with a priori thresholds of comparative fit index/Tucker-Lewis index ≥0.90 (preferably ≥0.95), root mean square error of approximation≤0.08 (preferably ≤0.06), and standardized root mean squared residual≤0.08; models failing these will be revised only based on theory and prespecified modification rules [36]. Internal consistency will be summarized using Cronbach α and McDonald ω, targeting α and ω values of 0.70 or higher for newly adapted scales and 0.80 or higher where feasible [28]. For multidimensional scales, α/ω will be reported per subscale; when a bifactor structure is indicated, we will report ωH (omega hierarchical).

Only scales demonstrating satisfactory psychometric properties (ie, strong validity and reliability) will be retained for subsequent hypothesis testing and regression analyses.

### Data Collection

Data collection will take place over 1 month. Quantitative data (via questionnaires) will be collected first, followed by qualitative data (including interviews, focus groups, and observations). System usage data (logs) will be collected retrospectively and prospectively during the study period.

### Data Management

Quantitative data will be collected electronically using the Open Data Kit platform, with forms programmed to include quality controls such as skip logic, input validation, and required fields. Encrypted data will be submitted daily to a secure central server. Qualitative audio recordings will be transcribed verbatim and fully anonymized by replacing personal identifiers with unique codes. All study data will be stored on a secure, password-protected server with access restricted to the research team and will be securely archived after the study, in accordance with relevant ethical guidelines.

### Implementation Feasibility and Mitigation Measures

We anticipated and proactively managed typical field constraints. To address geographic barriers across 72 often-remote facilities, data collection was scheduled before the rainy season, local enumerators were recruited, and contingency transport plans were prepared. Respondent availability was improved through pre-arranged appointments during low-volume periods and multiday visits at busier sites. Technology limitations were mitigated with offline-enabled forms, portable chargers, and standardized backup procedures. To minimize attrition, we used a census approach and engaged stakeholders throughout, yielding a 94.3% response rate. Qualitative saturation will be monitored in real time by two researchers conducting concurrent analysis, with flexible recruitment to target emerging gaps. Risks of social desirability bias are reduced through neutral-questioning training, use of external data collectors, and triangulation with system usage logs and observational notes. Timeline feasibility is supported by deploying 32 enumerators concurrently over 4 weeks with strong district-level coordination. Finally, coding reliability will be ensured through dual independent coding of 25% of transcripts, followed by consensus meetings to reconcile discrepancies and balance analytic rigor with interpretive depth.

### Statistical Analysis Plan

#### Quantitative Analysis

The statistical analysis will be conducted using Stata software (version 19). All hypothesis tests will be 2-tailed, with the significance level (α) set at .05. The analysis will commence only after the successful psychometric validation of all measurement scales.

##### Data Preparation and Cleaning

Before analysis, we will conduct rigorous data preparation. We will assess the extent and patterns of missingness; if missing data are minimal (<5%) and consistent with missing completely at random, we may use listwise or pairwise deletion for specific analyses. For more substantial or systematic missingness, we will apply multiple imputation to generate analysis-ready datasets for regression modeling. We will screen for univariate outliers (eg, via boxplots) and inspect bivariate relationships using scatterplots; influential observations will be reviewed and addressed case by case. Finally, we will evaluate distributional assumptions for all continuous variables (eg, implementation scores) using visual diagnostics (histograms, Q-Q plots) and formal tests (eg, Shapiro-Wilk).

##### Descriptive Analysis

A comprehensive descriptive analysis will be conducted to summarize the sample and key variables. First, the demographic and professional characteristics of the participants and health facilities will be summarized using frequencies and percentages. Second, the implementation scores for each MDE tool and the overall composite score will be summarized using key descriptive statistics, including the mean, standard deviation, median, IQR, and range. Finally, the determinant scores, derived from the scales measuring the CFIR constructs, will be summarized using descriptive statistics.

##### Inferential Analysis Plan

The inferential analysis will be structured to systematically test the study’s hypotheses.

A series of comparative tests will be conducted to test for variation in implementation (H1). Depending on the data distribution, Student *t* tests or Mann-Whitney *U* tests will be used to compare scores between two groups (eg, urban vs rural). In contrast, ANOVA or Kruskal-Wallis tests will compare scores across the 4 districts.

To explore associations with determinants (H2 and H3), Pearson correlation coefficients (for linear relationships) or Spearman correlation coefficients (for monotonic relationships) will be calculated to assess the strength and direction of the association between implementation scores and various determinant scores.

Given the hierarchical structure of the data—with health workers nested within health facilities (CSPS), which are nested within districts—linear mixed effects models (multilevel models) will be the primary analytical strategy to address H4 and overall heterogeneity. This approach correctly accounts for the nonindependence of observations and allows for variance partitioning across levels [37-39].

In the prespecified primary model, to guard against model over-complexity (J=72 facilities), a single parsimonious primary confirmatory model is prespecified: the composite MDE implementation score (facility-level mean) as outcome, with CFIR 2.0 Inner Setting and Individual Characteristics domain scores as Level-1 and Level-2 predictors, plus district as a fixed effect. A maximum of 10 to 12 facility-level predictors will be entered. All other model specifications are designated exploratory. The modeling will proceed sequentially through 3 steps.

In step 1, an initial model with no predictors, the unconditional (null) model, will be fitted to the main implementation outcome. This model will decompose the total variance into its components at the individual, facility, and district levels. The ICC will be calculated to quantify the proportion of total variance in MDE implementation attributable to clustering at the facility and district levels. This directly measures the heterogeneity of implementation across contexts.

In step 2, subsequently, a full multilevel model will be constructed by adding predictors at the individual level (eg, user age, professional experience, perceptions of the MDE), the facility level (eg, CSPS size, resource availability, leadership engagement), and the district level (eg, contextual factors).

In step 3, this final model will allow us to identify the individual, facility, and contextual determinants significantly associated with the degree of MDE implementation while appropriately controlling for the nested data structure. It will also reveal how much of the variation in implementation is explained by facility-related factors versus broader contextual factors.

##### Model Diagnostics

The final regression models will be rigorously checked for adherence to statistical assumptions, including linearity, normality of residuals, homoscedasticity, and the absence of significant multicollinearity (assessed using the variance inflation factor).

### Qualitative Analysis

#### Overall Approach and Rationale

The qualitative component of this study will be guided by a reflexive thematic analysis approach, following the 6-phase process described by Braun and Clarke [40,41]. This methodology was chosen for its flexibility and rigor in identifying, analyzing, and reporting patterns (or themes) within the data. It is particularly well-suited for exploring the complex, multilevel phenomena associated with the implementation of the MDE, allowing for a rich, detailed, and nuanced understanding of the “how” and “why” behind the quantitative findings.

#### Data Preparation

All audio recordings from semistructured interviews and focus group discussions will be transcribed verbatim. The transcripts will then be de-identified and anonymized to protect participant confidentiality; all personal names or unique identifiers will be replaced with alphanumeric codes. These anonymized transcripts, field observation notes, and open-ended survey responses will form the complete dataset for analysis.

#### Coding and Thematic Development Process

The analysis will be conducted using CAQDAS software (eg, NVivo) [42] and will follow a systematic, 6-phase thematic process. A hybrid coding strategy will be used, combining a deductive approach guided by our theoretical frameworks (CFIR 2.0 and NPT) with an inductive, data-driven approach to identify emergent themes [43].

#### Strategies for Ensuring Analytical Rigor and Trustworthiness

To ensure analytical rigor, we will implement several key strategies. These include triangulation across different data sources and methods, regular peer debriefing to enhance the credibility of coding and interpretation, thick description supported by participant quotations in the final report, and analyst reflexivity maintained through a detailed journal.

### Mixed Methods Integration

In line with this study’s sequential explanatory design (quantitative → qualitative) [18], the quantitative and qualitative strands will be integrated at multiple points. First, the quantitative results will directly inform the qualitative phase by guiding the purposive sampling of cases (eg, high- and low-performing sites) and the development of tailored interview and focus group guides. At the final interpretation stage, both datasets will be synthesized to generate meta-inferences [44], and team synthesis meetings will explicitly identify convergent and divergent findings. Joint displays linking facility-level quantitative implementation profiles with dominant qualitative themes will be constructed. Note that qualitative findings will inform the interpretation of quantitative associations but will not be used to respecify quantitative models post hoc [45]. Qualitative data will be used to explain and contextualize quantitative statistical patterns, while triangulation across the two strands will serve to confirm findings and strengthen the overall validity of the study’s conclusions [46].

### Ethical Considerations

This study received full ethical approval from the National Ethics Committee for Health Research of Burkina Faso (approval number 2023-06-136) and obtained additional authorizations from the Ministry of Health and relevant district authorities. Data will be immediately anonymized using unique codes to ensure confidentiality and stored on secure, password-protected servers with restricted access, and all consent forms will be stored separately. No financial compensation was provided; however, travel costs for focus group participants were reimbursed. Additionally, no personal identifiable information will be used in any publications.

Prior to participation, all individuals provided informed consent, either written or, for nonliterate participants, witnessed verbal consent, after being fully informed of the study’s objectives, their right to withdraw, and all confidentiality measures.

### Harms and Safety Monitoring

Given the minimal-risk, nonclinical nature of this study, a formal Data and Safety Monitoring Board has not been constituted. Any unexpected adverse events or breaches of confidentiality will be reported to the Ethics Committee for Health Research in accordance with national regulations.

## Results

This study was funded in June 2022 (Gates Foundation, Grant INV-056021) and is being conducted across 72 PHC facilities in 4 districts (Manga, Sapouy, Ténado, and Ziniaré) in Burkina Faso. Qualitative data collection commenced in November 2025 and was completed in January 2026. Quantitative data collection took place from January 2023 to October 2025. As of May 2026, qualitative data collection was completed, and quantitative analyses had commenced. Quantitative work currently includes data cleaning and preparation, descriptive summaries of participant and facility characteristics, and psychometric evaluation of all study scales (item performance, internal consistency, and construct validity). Multilevel modeling and qualitative thematic analysis are planned and will be initiated as data preparation milestones are met. Analytical activities are expected to conclude by July 2026. No outcome data are reported here, as this is a protocol. Finalized implementation scores and all model- and theme-level findings will be disseminated in the final study report and subsequent publications, expected in early 2027. Ethics approvals and consent procedures were completed before data collection, as detailed in the *Methods* section.

## Discussion

### Expected Outcomes

This study will produce two categories of outcomes: (1) empirical findings on the maturity and determinants of MDE implementation and (2) methodological products—validated, ready-to-use measurement scales and scoring procedures.

### Empirical Findings

This study will generate a nuanced portrait of implementation maturity across PHC facilities and districts, capturing adoption, fidelity, penetration, and perceived sustainability while quantifying between-facility heterogeneity through multilevel models (including ICCs and random-intercept variance). It will further produce an evidence map of multilevel determinants, anchored in CFIR 2.0 domains and complemented by NPT-informed patterns of normalization, integrating quantitative estimates with qualitative explanations. We will also document the extent to which MDE tools are institutionalized and routinely used within clinical and managerial workflows and examine their associations with PHC readiness and performance indicators (service delivery, stockouts, financial reporting, and data use).

Although the study uses a cross-sectional design at each measurement wave, the standardized and psychometrically validated instruments are purposefully designed for repeated administration. This means that implementation maturity can be measured at successive time points across the same facilities, enabling a repeated cross-sectional architecture that supports change-on-change analytical strategies—comparing shifts in contextual determinants with shifts in implementation scores across waves. This capacity to move beyond static, single-point associations toward identifying factors associated with improvement over time represents a methodological advance rarely achieved in digital health evaluations in SSA and positions the protocol as a foundation for prospective monitoring of the MDE’s trajectory.

The validated implementation measures enable a bidimensional strategic profiling of facilities along two complementary axes—implementation quality (how well the tools are used, as captured by psychometrically validated scores) and utilization (the extent to which available tools are actually integrated into routine practice). This dual profiling can generate a maturity typology (Success, Adoption Challenge, Deployment Challenge, Critical Zone) that translates complex statistical outputs into an actionable, intuitive classification directly usable by policymakers and district health management teams to target differentiated support strategies and monitor progress over successive evaluation cycles.

Together, these findings will culminate in actionable, context-specific recommendations to optimize and scale the MDE tailored to district and facility conditions.

### Methodological Products: Implementation Measurement Scales

This study will develop a suite of validated scales to quantify implementation outcomes for each MDE tool and for the ecosystem as a whole, following COSMIN guidelines (content and structural validity, reliability, measurement error, and hypothesis-testing construct validity). For each scale, we will report full psychometric evidence, including EFA/CFA results with model fit indices and item loadings, internal consistency (α and ω), and ωH where a bifactor structure is indicated—alongside measurement invariance checks across districts and staff cadres. Building on these metrics, we will construct a composite MDE implementation index with alternative weighting schemes (equal vs policy-informed weights), accompanied by prespecified sensitivity analyses. To facilitate uptake and reuse, user materials will be packaged for dissemination: Finalized instruments with item wording, a scoring codebook, and worked examples of score calculations will be provided for routine monitoring and future studies.

Collectively, these outputs will enable policymakers and program managers to define, measure, and track “implementation success” using transparent, psychometrically sound tools. The empirical findings will guide the prioritization of support (training, supervision, infrastructure) to accelerate the effective scale-up of the MDE.

### Expected Contributions of the Study

This study will advance implementation science for complex digital health interventions in resource-constrained settings by delivering a CFIR 2.0– and NPT-guided, mechanism-focused analysis of the multilevel determinants that shape adoption, integration into routine PHC workflows, and long-term institutionalization; generating generalizable insights across districts; and contributing methodological assets in the form of psychometrically validated implementation measurement scales (tool-specific and composite) with transparent scoring rules and invariance checks. Together, these outputs will provide an evidence-based roadmap for policymakers and practitioners—identifying leverage points such as leadership engagement, readiness, and support systems—to prioritize investments in training, supervision, infrastructure, and data use, thereby bridging the gap between the technological potential of the MDE and measurable, system-level impact.

### Generalizability and External Validity

Although this study is conducted in Burkina Faso, its findings are expected to have transferability to similar contexts. The study’s generalizability stems from three features: First, theoretical grounding in CFIR 2.0 and NPT—frameworks validated across diverse settings—ensures findings are interpreted through broadly applicable constructs rather than context-specific idiosyncrasies. Second, the validated measurement instruments produced by this study are intentionally designed for adaptability; items reference generalizable implementation constructs (eg, “perceived ease of use,” “organizational readiness”) rather than Burkina Faso–specific details. Third, the multilevel analytical approach explicitly models contextual variation (between districts, facilities, individuals), allowing identification of which findings are setting-dependent versus universal.

That said, we acknowledge limitations to generalizability: The MDE’s specific configuration (choice of 9 tools, CommCare/DHIS2 platforms) may differ from digital ecosystems elsewhere, Burkina Faso’s health system structure (CSPS as the PHC backbone, the gratuité policy) may not mirror other countries, and infrastructure constraints in the study districts may be more or less severe than other SSA settings.

### Recommendation for Adaptation

Researchers and policymakers in other contexts should adapt the study’s approach—integrated ecosystem evaluation using CFIR/NPT with validated scales—rather than directly applying the specific findings without contextual consideration. Our instruments and methods provide a replicable template while acknowledging that determinants and thresholds of “implementation success” may vary by setting.

### Potential Implications of Expected Results

The expected results carry implications from near-term operational improvements to long-term health system strengthening. Identifying the factors most strongly associated with implementation outcomes would justify targeted actions—such as upgrading infrastructure, refining workflows, and institutionalizing ongoing professional development—to deliver a more robust and responsive MDE rollout. Over time, sustained use of the MDE is poised to enhance data quality, streamline clinical and administrative processes, and improve accountability and resource use. Collectively, these gains can translate into measurable improvements in access to and quality of essential PHC services, inform scalable strategies for digital health investment, and contribute to system resilience and equity in resource-constrained settings.

### Strengths of the Protocol

This protocol has several strengths that enhance its scientific rigor and policy relevance. First, the sequential explanatory mixed methods design (quantitative → qualitative) integrates the breadth of quantitative findings with the descriptive depth of qualitative inquiry, enabling not only the measurement of implementation maturity but also the explanation of the mechanisms underlying observed patterns. Second, the explicit dual grounding in CFIR 2.0 and NPT provides complementary analytical lenses—CFIR 2.0 to identify *what* multilevel factors shape implementation and NPT to illuminate *how* digital tools become normalized (or fail to become normalized) within clinical and managerial workflows. Third, the multilevel data collection strategy captures perspectives from providers, facility managers, community health workers, and health service users across 72 facilities and 4 districts, enabling robust triangulation and sufficient statistical power for multilevel modeling. Fourth, the rigorous psychometric development and validation of all implementation measurement scales—including EFA and CFA, reliability assessment, and measurement invariance testing across districts and staff cadres—ensure that observed differences reflect genuine variation in implementation rather than measurement artifacts. Finally, the pragmatic orientation of the study—evaluating the MDE “as deployed” rather than under idealized conditions—ensures that findings are directly applicable to the real-world contexts in which scale-up decisions will be made.

### Potential Limitations of the Protocol

This protocol has several potential limitations. First, reliance on self-reported data introduces risk of social desirability bias; we mitigate this through intensive enumerator training, neutral interviewing, and strict anonymity procedures. Second, the cross-sectional, observational survey design is suitable for characterizing implementation at a point in time but precludes causal inference; all multilevel model estimates reflect statistical associations rather than causal effects. The subsequent qualitative phase is intended to contextualize and explain observed associations. Third, generalizability may be constrained to settings with comparable health system structures and digital readiness. Finally, logistical challenges—such as reaching remote sites and accommodating busy clinical schedules—may affect recruitment and timing, necessitating flexible fieldwork plans and persistent follow-up.

### Dissemination Strategies for Future Results

We will pursue a multi-audience strategy. For policymakers and program managers, comprehensive evaluation reports and concise policy briefs will be delivered to the Ministry of Health, district authorities, and funding partners. For frontline providers and communities, we will convene interactive feedback workshops to review the results and discuss the practical implications. To contribute to the global evidence base, we will publish in reputable peer-reviewed journals (including this protocol) and present at major international health conferences. To engage the public and media, we will produce plain-language summaries and fact sheets for broad distribution. Where permissible, we will share an anonymized dataset under a Data Use Agreement to support secondary analyses and reproducibility.

### Conclusions

This protocol outlines a pivotal study to evaluate the implementation of a national digital health ecosystem in Burkina Faso. By generating robust evidence on implementation maturity, fidelity, and their key determinants, the research will directly inform optimization of the MDE and guide future digital health strategies. Ultimately, these insights are expected to support stronger, more equitable PHC across SSA.

## Supplementary material

10.2196/86135 Checklist 1 SPIRIT (Standard Protocol Items: Recommendations for Interventional Trials) 2013 (adapted) checklist for an implementation study; TIDieR (Template for Intervention Description and Replication) checklist for the Minimal Digital Ecosystem (MDE) intervention.

## Abbreviations

ASBC: Agent de Santé à Base Communautaire (community-based health worker)
CFA: confirmatory factor analysis
CFIR: Consolidated Framework for Implementation Research
COSMIN: Consensus-Based Standards for the Selection of Health Measurement Instruments
CSPS: Centre de Santé et de Promotion Sociale (Primary Health and Social Promotion Center)
DEFF: design effect
ECD: Équipe Cadre de District (District Health Management Team)
EFA: exploratory factor analysis
ICC: intracluster correlation coefficient
ICP: Infirmier Chef de Poste (Head Nurse of Post)
MDE: Minimal Digital Ecosystem
mHealth: mobile health
NPT: Normalization Process Theory
PHC: primary health care
REC-PCIME: Registre Électronique de Consultation - Prise en Charge Intégrée des Maladies de l’Enfant (Electronic Consultation Register - Integrated Management of Childhood Illness)
SSA: sub-Saharan Africa
TIDieR: Template for Intervention Description and Replication
