# Supplementary material for: Strengthening Primary Care With a Minimal Digital Ecosystem in Burkina Faso: Protocol for a Pragmatic Mixed Methods Implementation Study
Source: JMIR Res Protoc. 2026 May 22;15:e86135. doi: 10.2196/86135 (PMC13197158; doi:10.2196/86135)
Supplement: Checklist 1 [file resprot-v15-e86135-s001.docx]

### **Appendix 1: SPIRIT (Standard Protocol Items: Recommendations for Interventional Trials) 2013 Checklist**

**Note:** This study is a pragmatic, observational, mixed-methods evaluation of an existing implementation rather than an interventional trial. Therefore, items related to intervention allocation, blinding, and randomization are not applicable (N/A). The checklist is completed to reflect the protocol's structure for transparency and rigor.

| Section/Topic | Item No. | Checklist Item | Location in Manuscript |
| --- | --- | --- | --- |
| Administrative Information |  |  |  |
| Title | 1 | Descriptive title identifying the study design, population, intervention, and, if applicable, trial acronym. | Title Page |
| Trial registration | 2a | Trial identifier and registry name. If not yet registered, name of intended registry. | N/A. This is an observational implementation study, not a clinical trial requiring registration. |
|  | 2b | All items from the World Health Organization Trial Registration Data Set. | N/A |
| Protocol version | 3 | Date and version identifier. | The protocol is dated September 25, 2025. |
| Funding | 4 | Sources and types of financial, material, and other support. | Funding |
| Roles and responsibilities | 5a | Names, affiliations, and roles of protocol contributors. | Authors' Contributions; Acknowledgements |
|  | 5b | Name and contact information for the trial sponsor. | Funding. The study is funded by the Gates Foundation. |
|  | 5c | Role of study sponsor and funders, if any, in study design; collection, management, analysis, and interpretation of data; writing of the report; and the decision to submit the report for publication, including whether they will have ultimate authority over any of these activities. | Funding |
|  | 5d | Composition, roles, and responsibilities of the coordinating centre, steering committee, endpoint adjudication committee, data management team, and other individuals or groups overseeing the trial, if applicable. | Authors' Contributions; Acknowledgements |
| Introduction |  |  |  |
| Background and rationale | 6a | Description of research question and justification for undertaking the trial, including a summary of relevant studies. | Introduction; Justification for the revised evaluation objectives |
|  | 6b | Explanation for choice of comparators. | N/A. The initial quasi-experimental design with control districts was compromised by intervention contamination . The study now assesses varying implementation levels across all sites. |
| Objectives | 7 | Specific objectives or hypotheses. | Study goals and objectives; Hypotheses |
| Trial design | 8 | Description of trial design including type of trial, allocation ratio, and framework. | Study Design |
| Methods: Participants, interventions, and outcomes |  |  |  |
| Study setting | 9 | Description of study settings where data will be collected. Reference to where list of sites can be obtained. | Study Setting |
| Eligibility criteria | 10 | Inclusion and exclusion criteria for participants. | Eligibility and Exclusion Criteria |
| Interventions | 11a | Interventions for each group with sufficient detail to allow replication, including how and when they will be administered. | Intervention Description |
|  | 11b | Criteria for discontinuing or modifying allocated interventions for a given trial participant. | N/A. The study is evaluating an existing real-world implementation. |
|  | 11c | Strategies to improve adherence to intervention protocols, and any procedures for monitoring adherence. | N/A. The study's primary goal is to |
|  | 11d | Relevant concomitant care and interventions that are permitted or prohibited during the trial. | N/A. |
| Outcomes | 12 | Primary, secondary, and other outcomes, including the specific measurement variable, analysis metric, method of aggregation, and time point for each outcome. | Outcomes and Data Collection |
| Methods: Assignment of interventions |  |  |  |
| Allocation | 16a-c | N/A. | This is not a randomized trial. |
| Blinding | 17a-b | N/A. | This is an unblinded observational study. |
| Methods: Data collection, management, and analysis |  |  |  |
| Data collection methods | 18a | Plans for assessment and collection of outcome, baseline, and other trial data, including any related processes to promote data quality. | Measurement Instruments; Scale Development and Validation; Data Collection; Data Management |
|  | 18b | Plans to promote participant retention and complete follow-up, including list of any outcome data to be collected for participants who discontinue or deviate from intervention protocols. | N/A. This is a cross-sectional survey design. |
| Data management | 19 | Plans for data entry, coding, security, and storage, including any related processes to promote data quality. | Data Management |
| Statistical methods | 20a | Statistical methods for analysing primary and secondary outcomes. | Statistical Analysis Plan |
|  | 20b | Methods for any additional analyses (e.g., subgroup and adjusted analyses). | Statistical Analysis Plan |
|  | 20c | Definition of analysis population relating to protocol non-adherence and any statistical methods to handle missing data. | Statistical Analysis Plan |
| Methods: Monitoring |  |  |  |
| Data monitoring | 21a | Composition of data monitoring committee (DMC); summary of its role and reporting structure; statement of whether it is independent from the sponsor and competing interests. | Harms and Safety Monitoring |
|  | 21b | Description of any interim analyses and stopping guidelines. | N/A. |
| Harms | 22 | Plans for collecting, assessing, reporting, and managing solicited and spontaneously reported adverse events and other unintended effects of trial interventions or trial conduct. | Harms and Safety Monitoring |
| Auditing | 23 | Frequency and procedures for auditing trial conduct, if any, and whether the process will be independent from investigators and the sponsor. | Not specified. |
| Ethics and dissemination |  |  |  |
| Research ethics approval | 24 | Plans for seeking research ethics committee/institutional review board approval. | Ethical Considerations |
| Protocol amendments | 25 | Plans for communicating important protocol modifications to relevant parties. | Not specified, but this would be managed via the approving ethics committee. |
| Consent or assent | 26a | Who will obtain informed consent or assent from potential trial participants or authorized surrogates, and how. | Consent to Participate |
|  | 26b | Additional consent provisions for collection and use of participant data and biological specimens in ancillary studies, if applicable. | N/A. No biological specimens collected. Data sharing is covered. |
| Confidentiality | 27 | How personal information about potential and enrolled participants will be collected, shared, and maintained in order to protect confidentiality before, during, and after the trial. | Data Management; Consent to Participate |
| Declaration of interests | 28 | Financial and other competing interests for principal investigators for the overall trial and each study site. | Conflicts of Interest |
| Access to data | 29 | Statement of who will have access to the final trial dataset, and disclosure of contractual agreements that limit such access for investigators. | Data Availability Statement |
| Ancillary and post-trial care | 30 | Provisions, if any, for ancillary and post-trial care, and for compensation to those who suffer harm from trial participation. | N/A. This is a minimal-risk, non-clinical study. |
| Dissemination policy | 31a | Plans for investigators and sponsor to communicate trial results to participants, healthcare professionals, the public, and other relevant groups, including any publication restrictions. | Dissemination Strategies for Future Results |
|  | 31b | Authorship eligibility guidelines and any intended use of professional writers. | Authors' Contributions |
|  | 31c | Plans, if any, for granting public access to the full protocol, participant-level dataset, and statistical code. | Dissemination Strategies; Data Availability Statement |

### **Appendix 2: TIDieR (Template for Intervention Description and Replication) Checklist**

| Item No. | Item | Description |
| --- | --- | --- |
| 1. BRIEF NAME | Provide the name or a phrase that describes the intervention. |  |
| 2. WHY | Describe any rationale, theory, or goal of the elements essential to the intervention. | The goal is to address fragmentation of digital tools and strengthen primary healthcare by integrating multiple point-of-care and management applications. The |
| 3. WHAT (Materials) | Describe any physical or informational materials used in the intervention, including those provided to participants or used in intervention delivery or training. | The MDE is a software-based intervention comprised of nine core digital tools |
| 4. WHAT (Procedures) | Describe each of the procedures, activities, and/or processes used in the intervention, including any enabling or support activities. | The procedures involve the routine use of the MDE tools by healthcare staff to perform their daily functions: |
| 5. WHO PROVIDED | For each category of intervention provider (e.g., psychologist, nursing assistant), describe their expertise, background, and any specific training given. | The MDE was introduced as a national initiative led by the Ministry of Health and its partners. While not detailed in the protocol, training on the use of MDE tools was provided to the relevant cadres of health personnel as part of the implementation process. |
| 6. HOW | Describe the modes of delivery (e.g., face-to-face, internet) and the settings in which the intervention was delivered. | The intervention is delivered digitally through specific software applications installed on tablets, mobile phones, and computers at health facilities and in the community. |
| 7. WHERE | Describe the type of location(s) where the intervention occurred, including any necessary infrastructure or relevant features. | The intervention is implemented in |
| 8. WHEN and HOW MUCH | Describe the number of times the intervention was delivered and over what period of time, including the number of sessions, their schedule, and their duration, intensity, or dose. | The MDE tools are designed for continuous and routine use as part of the daily workflow of healthcare providers and managers. The "dose" is not a fixed number of sessions but rather the degree of adoption, penetration, and integration into routine practice, which is a primary outcome this study aims to measure. |
| 9. TAILORING | If the intervention was planned to be personalized, titrated, or adapted, then describe what, why, when, and how. | The protocol does not describe any specific tailoring of the MDE intervention. The study evaluates the implementation of a standardized suite of tools deployed across all sites. |
| 10. MODIFICATIONS | If the intervention was modified during the course of the study, describe the changes (what, why, when, and how). | N/A. This protocol describes an evaluation of an existing implementation; it does not involve modifying the intervention. |
| 11. HOW WELL (Planned) | If intervention fidelity was assessed, describe how and by whom, and if any strategies were used to maintain or improve fidelity. | Yes, assessing fidelity is a core objective. |
| 12. HOW WELL (Actual) | If intervention fidelity was assessed, describe the extent to which the intervention was delivered as planned. | N/A. This is a study protocol; data on actual fidelity have not yet been fully collected or analyzed. |

### **Appendix 3: TIDieR (Template for Intervention Description and Replication) Checklist for each tool**

| **Tool Name** | **Why (Rationale & Objectives)** | **What (Functionalities & Materials)** | **How, Where & Who (Delivery & Users)** | **When, How Much & How Well (Dose & Implementation Fidelity)** |
| --- | --- | --- | --- | --- |
| REC-PCIME | This tool was developed to improve healthcare provider adherence to the national Integrated Management of Childhood Illnesses (IMCI) protocol. It aims to standardize consultations, reduce diagnostic errors, and create a robust electronic database for epidemiological surveillance. | REC-PCIME is a job aid and data collection tool built on the CommCare platform. It guides providers step-by-step through the IMCI protocol. It includes automated calculations for medication dosage and provides clinical decision support. The primary material is an Android tablet with the REC-PCIME application. | The tool is used by healthcare workers (nurses, doctors) during pediatric consultations within Primary Health and Social Promotion Centers (CSPS). The core functionality was standardized and not tailored for individual facilities. | Dose: The tool is intended for use during every consultation with a child under five. Implementation: Prior to deployment, all relevant staff received a one-time, 3-day training workshop conducted by certified trainers from Terre des Hommes. Post-training fidelity is supported by monthly on-site supportive supervision visits from district supervisors. A process for managing software updates is in place. |
| REC-Maternité | This tool aims to improve the quality and continuity of maternal and newborn care by standardizing procedures according to national protocols. It seeks to create a longitudinal electronic record for each mother-newborn dyad. | Built on CommCare, this application features modules for Antenatal Care (ANC), Delivery, Postnatal Care (PNC), etc. It provides checklists and risk alerts. The materials consist of an Android tablet with the REC-Maternité software. | Midwives and other trained health workers use the tool during maternal and newborn health consultations at CSPS facilities. Clinical workflows are standardized across all sites. | Dose: Intended for use at every maternal/newborn visit (ANC, delivery, PNC). Implementation: Staff received a 3-day modular training from MOH-certified trainers. Fidelity is maintained through bi-monthly data quality audits by the district team and a user-led community of practice. |
| E-Feuille de Soins (E-Care Sheet) | The goal is to replace the inefficient, paper-based system for the national free healthcare (gratuité) policy. It aims to reduce administrative burden on providers, minimize billing errors, and accelerate the reimbursement process. | This application allows providers to digitally complete the individual patient care sheet (Fiche Individuelle de Soins), detailing services and medications provided. The digital record serves as an electronic claim. It is deployed on Android tablets. | It is used at the point of care by prescribing health workers within CSPS facilities immediately after a consultation covered by the gratuité policy. The form is standardized as per national policy. | Dose: Used for every patient care episode covered by the gratuité scheme. Implementation: A 1-day training focused on the administrative and financial workflows was provided. High fidelity is ensured by its mandatory role as the sole mechanism for facility reimbursement. |
| NetSIGL 2.0 | This platform addresses the critical challenge of stock-outs of essential medicines. It aims to improve supply chain management by providing real-time visibility into stock levels and consumption data. | NetSIGL 2.0 is an electronic logistics management information system (eLMIS) on the DHIS2 platform. It allows pharmacy managers to track inventory, report consumption, and place orders electronically. It is accessible on Android tablets and computers. | The tool is used primarily by pharmacy managers within the CSPS storerooms. It is a standardized national tool. | Dose: Requires weekly data entry for key stock levels and a comprehensive report monthly. Implementation: A 2-day training on logistics principles and the software was provided. Fidelity is supported by a "buddy system" and monthly data review meetings at the district level. |
| E-Flux Financier | This tool was designed to address challenges of financial opacity and inefficient management. Its primary goal is to enhance transparency, improve accountability, and build the capacity of local managers for sound financial stewardship. | E-Flux Financier is an application for real-time tracking of all financial transactions. It automates the generation of standardized monthly financial reports. The key material is an Android tablet with the dedicated software. | The tool is used by the person responsible for financial management at the CSPS. Data is aggregated on a central dashboard for oversight. | Dose: Daily entry of financial transactions is expected. Implementation: A 2-day specialized training was provided by the Clinton Health Access Initiative. Fidelity is maintained through monthly remote coaching and mentorship sessions with financial supervisors. |
| mHealth Communautaire | This tool aims to extend the reach of the primary healthcare system into communities, empower Community-Based Health Workers (ASBCs) with standardized protocols, and ensure systematic community data capture. | Built on the CommCare platform, this application is deployed on Android mobile phones. It contains simplified diagnostic guidelines, health promotion messaging, and tools for tracking patients. | The tool is used by ASBCs on their phones during daily household visits and community activities. Protocols are standard, but activities are chosen based on community needs. | Dose: Intended for daily use during community activities. Implementation: ASBCs received a 3-day initial training, with annual refreshers planned. Fidelity is supported via a monthly supervision meeting with a designated supervisor at their local CSPS. |
| E-Quality | The tool is designed to support Burkina Faso's national health facility certification process. It aims to replace paper-based audits with an efficient digital system to foster a culture of continuous quality improvement. | This is a DHIS2-based platform that houses digital checklists for internal (self-assessment) and external quality audits against national standards. It automatically calculates quality scores. It is accessible on both Android tablets and computers. | Used by facility managers for internal audits and by district/regional auditors for external assessments at CSPS. The checklists are nationally standardized. | Dose: Intended for quarterly internal self-audits and annual external audits. Implementation: A 1-day training on the national quality standards and the tool was provided. High fidelity is driven by the tool's institutional link to the official facility certification process. |
| E-Gratuité | This platform aims to improve the management, transparency, and accountability of the national gratuité scheme by providing reliable data on services, invoicing, and payments. | A DHIS2-based platform, E-Gratuité is the official system for collecting all data related to the policy, including invoices, payment tracking, and consumption data. It is used on tablets and computers. | Used by facility managers for invoicing and by district managers for data validation and monitoring. It is a mandatory, standardized national reporting tool. | Dose: Used for compiling and submitting mandatory monthly invoices and reports. Implementation: A 1-day training on reporting requirements was provided. Adoption and fidelity are ensured as it is the sole channel for facilities to receive reimbursement for free services. |
| The MDE Dashboard | The Dashboard was developed to solve the problem of data fragmentation. It aims to provide managers at all levels with a single, integrated view of key performance indicators to support timely, evidence-based decision-making. | It is a web-based digital interface that aggregates, analyzes, and visualizes selected indicators from all other MDE tools. Data is updated dynamically and automatically. It was developed using Python by Cooper Smith. | Accessed via a web browser on computers or mobile devices by health managers at all levels. The tool is highly tailored, with different user roles having customized views. | Dose: Intended for at least weekly review by managers. Implementation: A half-day orientation session focused on data interpretation and use was provided. Its utility and use are dependent on the data quality of the feeder systems. A user-centered design process was key to ensuring relevance. |
